# P-13. Awareness, Attitudes, Knowledge and Barriers Related to Adult Pneumococcal Vaccination among Internal Medicine Residents and Fellows in University Hospital, Thailand

**DOI:** 10.1093/ofid/ofae631.223

**Published:** 2025-01-29

**Authors:** Thanasate Roongjiraroj, Pawat Phuensan

**Affiliations:** King Chulalongkorn Memorial Hospital, Phramongkutklao College of Medicine, Bangkok, Krung Thep, Thailand; King Chulalongkorn Memorial Hospital, Krung Thep, Thailand

## Abstract

**Background:**

Pneumonia is the leading cause of global mortality with *Streptococcus pneumoniae* as a leading pathogen in high-risk adults. Although vaccination is crucial for preventing pneumococcal infections, its uptake remains suboptimal in Thailand. Internists, pivotal in managing such patients, are at the forefront of promoting vaccination. Therefore, this study aimed to explore factors related to physicians’ pneumococcal vaccine administration among high-risk adults in university hospital in Thailand.

**Figure:**
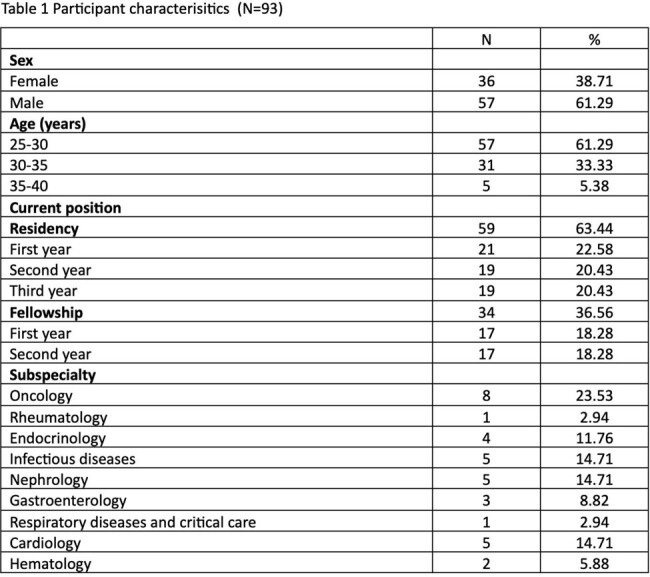

**Methods:**

This web-based survey cross-sectional study was conducted at King Chulalongkorn Memorial Hospital, Bangkok, Thailand (university hospital). Participants were residents and fellows in Department of Medicine. The questionnaire included demographic data and vaccine-related inquiries in various aspects including awareness, attitudes, and knowledge towards pneumococcal vaccination.

**Figure:**
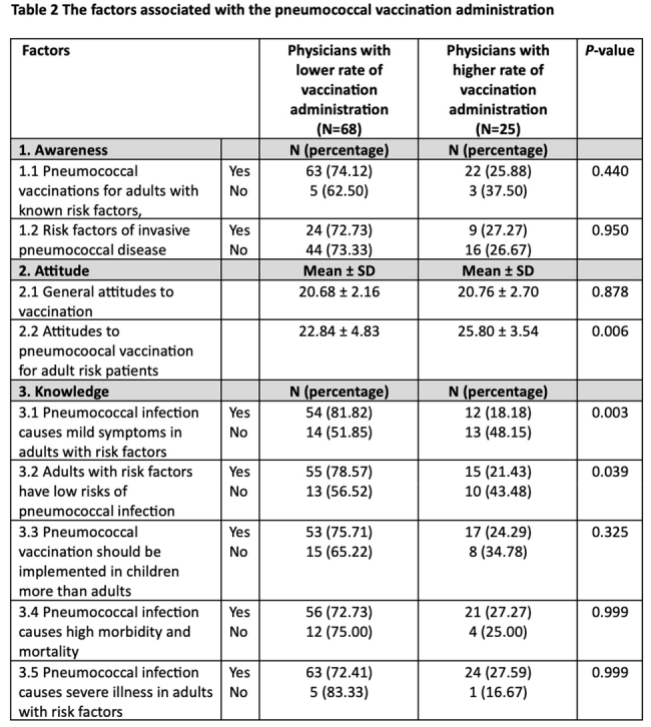

**Results:**

The study involved 93 participants. Demographic data was presented in table 1. Awareness of pneumococcal vaccination was generally high and attitudes were positive. Overall, participants showed a good knowledge of pneumococcal infection, but participants with lower rate of vaccination administration had significant lower knowledge than participants with higher rate of vaccination administration (table 2). Key barriers to vaccination included limited access to advice and recording systems, cost issues, and a lack of specialized consultation. Potential quality improvement strategies were electronic vaccination record, easy-to-access guideline, and vaccine consultation clinic.

**Conclusion:**

This study emphasizes the importance of addressing barriers like limited access to vaccination advice and recording systems, cost issues, and a lack of specialized consultation. Implementing targeted interventions and raising knowledge and awareness of vaccination are key steps toward reducing the burden of pneumococcal infections. These results would lead to improvement of residency and fellowship training program and vaccination practice in our hospital.

**Disclosures:**

**Pawat Phuensan, MD, MSc**, GSK: Grant/Research Support|GSK: Honoraria|MSD: Honoraria|Pfizer: Grant/Research Support|Pfizer: Honoraria|Siam Pharmaceutical: Grant/Research Support|Takeda: Honoraria

